# Commissioning of a respiratory gating system involving a pressure sensor in carbon‐ion scanning radiotherapy

**DOI:** 10.1002/acm2.12463

**Published:** 2018-11-01

**Authors:** Hideyuki Mizuno, Osami Saito, Minoru Tajiri, Taku Kimura, Daigo Kuroiwa, Toshiyuki Shirai, Taku Inaniwa, Mai Fukahori, Kentaro Miki, Shigekazu Fukuda

**Affiliations:** ^1^ National institute of Radiological Sciences, QST Chiba Japan; ^2^ Hiroshima University Hospital Hiroshima Japan

**Keywords:** pressure sensor, quality assurance in radiotherapy, respiratory gating

## Abstract

This study reports the commissioning methodology and results of a respiratory gating system [AZ – 733 V/733 VI (Anzai Medical Co., Japan)] using a pressure sensor in carbon‐ion scanning radiotherapy. Commissioning includes choosing a location and method for pressure sensor installation, delay time measurement of the system, and the final flow test. Additionally, we proposed a methodology for the determination of a threshold level of generating an on/off gate for the beam to the respiratory waveform, which is important for clinical application. Regarding the location and method for installation of the pressure sensor, the actual person's abdomen, back of the body position, and supine/prone positioning were checked. By comparing the motion between the pressure sensor output and the reference LED sensor motion, the chest rear surface was shown to be unsuitable for the sensor installation, due to noise in the signal caused by the cardiac beat. Regarding delay time measurement of the system, measurements were performed for the following four steps: (a). Actual motion to wave signal generation; (b). Wave signal to gate signal generation; (c). Gate signal to beam on/off signal generation; (d). Beam on/off signal to the beam irradiation. The total delay time measured was 46 ms (beam on)/33 ms (beam off); these were within the prescribed tolerance time (<100 ms). Regarding the final flow test, an end‐to‐end test was performed with a patient verification system using an actual carbon‐ion beam; the respiratory gating irradiation was successfully performed, in accordance with the intended timing. Finally, regarding the method for determining the threshold level of the gate generation of the respiration waveform, the target motion obtained from 4D‐CT was assumed to be correlated with the waveform obtained from the pressure sensor; it was used to determine the threshold value in amplitude direction.

## 1 INTRODUCTION

A three‐dimensional pencil beam scanning irradiation system using a carbon‐ion beam was developed at the Heavy Ion Medical Accelerator in Chiba (HIMAC), National Institute of Radiological Sciences (Chiba, Japan).^1^, ^2^, ^3^ Clinical application started in 2011; however, it was limited to cases without respiratory motion in the initial stage.^4^ Pencil beam scanning is more sensitive to organ motion than conventional broad‐beam irradiation.^5^, ^6^, ^7^ The interplay effect between the scanning motion and target motion leads to hot and/or cold spots in dose distribution over the target volume.^8^, ^9^ Therefore, a combination of a rescanning technique and a gated irradiation method was used to mitigate the induction of hot/cold spots.^10^, ^11^ For the gated irradiation method, it is necessary to use the respiration signal. There are two techniques for obtaining a respiratory signal, external and internal.^12^ This study addressed only the external respiratory signal, which is routinely used in current clinical treatment at our center.

To control beam extraction from the accelerator, we adopted a commercial respiratory gating system with a pressure sensor, which is rarely used for radiation therapy.^13^ A laser distance meter is popularly used to monitor the motion of a patient's body surface to acquire respiratory signals in particle beam therapy.^14^ However, the laser distance meter installed on the patient couch interferes with the tool used to immobilize the patient. The pressure sensor is superior from the viewpoint of clearance, and can be easily attached to the patient by inserting it between the patient and the immobilization shell. Thus far, there have been no reports describing commissioning processes involving a pressure sensor for radiation therapy, especially in particle therapy.

This report describes the commissioning methodology, including assessment of system accuracy and clinical safety, for a pressure sensor in carbon‐ion scanning radiotherapy. Commissioning included the choice of location and method of sensor installation, the measurement of system gating delay time, and final flow test. Dosimetric measurements were beyond the scope of this study, as there were reported previously.^10^ In addition, we propose a methodology to determine the threshold level to generate an on/off gate for the beam.

## 2 MATERIALS AND METHODS

### 2.A Respiratory gating system

**Figure 1:**
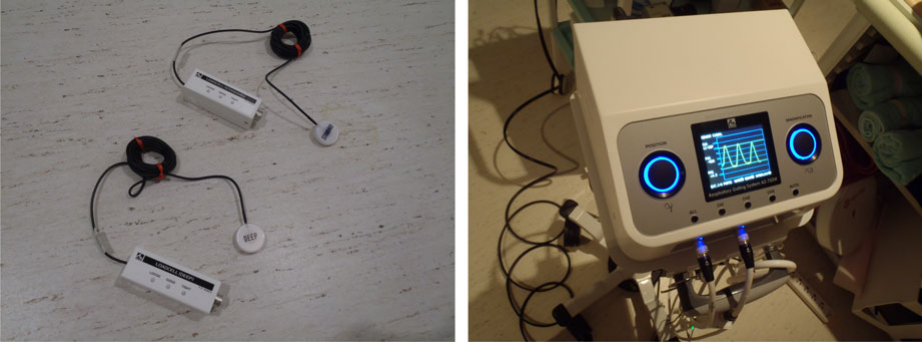
Left panel: Pressure sensors (two types with different responses). Right panel: Sensor port located beside the patient couch.

The respiratory gating system used in this study was the AZ‐733V/733VI (Anzai Medical Co., Tokyo, Japan). The system consists of pressure sensors (load cell, dimensions 30 mm‐diameter, 9.5‐mm thickness; two types with different response modes [Fig. 1]), a sensor port located beside the couch bed that is connected to the sensors via a pre‐amp, a repeater located in the operator room, and a control PC. In addition, a belt was used to attach the pressure sensor to the patient during installation. Using this belt, the respiratory signal was acquired. The original respiratory signal acquired from the pressure sensor was transferred to the control PC via the sensor port along with timing information to generate a respiratory waveform. The control PC generated a beam on gate signal depending on the intended amplitude of the waveform, otherwise known as the threshold level.

### 2.B Location and sensor installation method

**Figure 2:**
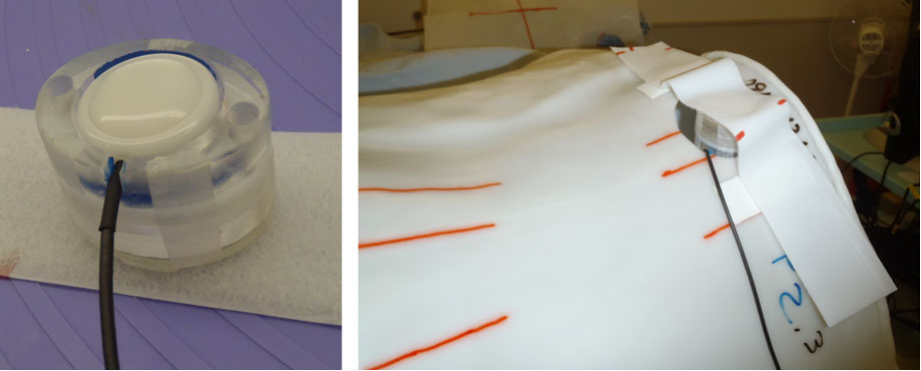
Left panel: An in‐house‐manufactured sensor holder. Right panel: The holder mounted to the patient immobilization shell.

**Figure 3:**
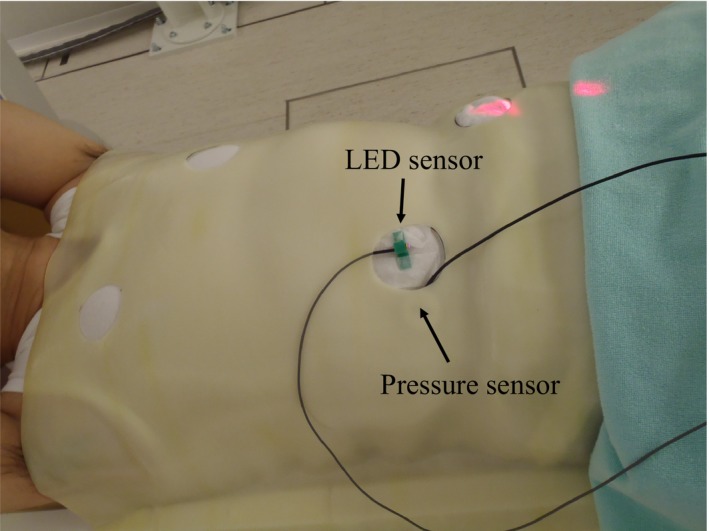
Test signal acquisition using both a light‐emitting diode (LED) sensor and a pressure sensor. Both sensors were installed side by side.

Because it was not easy to use the belt‐type sensor holder (provided by the vendor) together with the immobilization tool, which is a low‐temperature thermoplastic shell that wraps around the entire abdomen of the patient, we first considered how to attach the pressure sensor to the surface of the patient. Several locations and methods of installing the sensor were tested on clinical staff members, in a manner consistent with typical clinical situations. Both young staff and senior staff members cooperated in this test to imitate variation of the patient age. First, an immobilization shell was fabricated to resemble a patient. Next, a hole that did not interfere with the treatment beam was made to insert the sensor or to attach the in‐house‐manufactured sensor holder (Fig. 2). The respiratory signal acquired from the pressure sensor was then compared with a signal from the motion of a light‐emitting diode (LED) placed on the surface of the body as a reference (Fig. 3). To compare the overall shape including the amplitude and phase, both waveforms were superimposed. The LED sensor (Toyonaka Kenkyujo Co., Osaka, Japan) has long been used for carbon‐ion passive radiotherapy and quality was verified.^15^

As shown in Fig. 3, the pressure and LED sensors were installed side by side (the pressure sensor was under the shell.). A respiratory waveform was acquired by each system. Because the two systems are completely independent, to match the time axis the characteristic peak of the waveform was made externally at the same time for both systems. To accomplish this, the LED was manually interrupted instantaneously as soon as the hand hit the pressure sensor. The scale of the waveform amplitude was adjusted so that the average values of several peaks after the characteristic peak were consistent.

### 2.C Measurement of system delay time

The delay times in each conversion step of the system were measured as follows:

1. Actual motion to wave signal generation.
2. Wave signal to gate signal generation.
3. Gate signal to beam on/off signal generation.
4. Beam on/off signal to the beam irradiation.

These delay times were added together to obtain a final delay time. In step 1, a calibrated laser range meter was used as a reference. The pressure sensor was placed on a board and the surface of the sensor was targeted by the laser range meter, meaning that the laser pointer from the laser sensor was situated exactly on the surface of the pressure sensor. The outputs of the pressure sensor and the laser range meter were connected to an oscilloscope. A rapid shock (delivered by tapping the board) was applied to the sensor, resulting in both pressure change and range change. The difference between the responses measured on the oscilloscope for the two input signals was considered the delay time. In step 2, the difference between the time when the waveform signal reached the intended amplitude (50% of the maximum amplitude in this case) and the time of gate signal generation was measured. To mimic the patient's respiratory motion, a dynamic thorax phantom (CIRS Inc., Norfolk, VA, USA) was used with a sine‐wave pattern. The waveform signal was acquired from the sensor output and the gate signal from the respiratory system output. Both outputs were inputted to the oscilloscope to measure the delay time. In step 3, the delay time between the gate signal from the respiratory gating system and the beam on/off signal generated by the irradiation system was measured using an oscilloscope. In step 4, the delay time between the beam on/off signal and the beam irradiation timing was measured using data logger record connected to the analogue output of dose monitor.

### 2.D Final flow test

As in actual patients, a final flow test — which is often referred to as an “end‐to‐end” test — was performed using a treatment management system (connected to a recording and verification system) with a phantom. Patient motion was again mimicked by a dynamic thorax phantom. The waveform of a real patient was used as input for the dynamic phantom control. The flow test included the following items: irradiation mode in the R&V system set to “external respiratory gating” in accordance with the planning system, the enabling of a regular patient alignment sequence, successful acquisition of respiratory signal, the enabling of gate generation, the enabling of irradiation with the intended timing, the enabling of rescanning, functioning of interlocks, and the ability to save the respiratory signal/gate/beam on information.

### 2.E Determination of threshold level to generate gate signal in clinical situations

**Figure 4:**
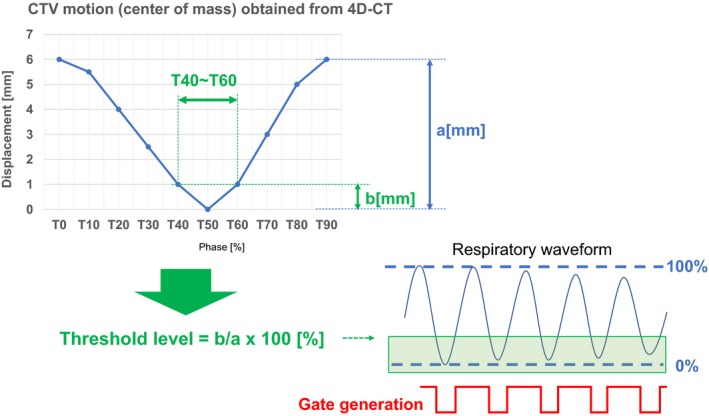
Method for determining the threshold gate generation for beam on/off. The threshold level was derived from the tumor (clinical target volume;CTV) motion obtained from four‐dimensional computed tomography (4D‐CT) information.

Finally, we added a consideration for determining the threshold level to generate gate signals in clinical situations. This topic is not a mechanical quality assurance item of the system; however, it is a very important aspect for initiating the clinical use of the system. To determine the threshold level in the amplitude direction of the respiratory waveform in order to generate the beam's on/off gate signal, clinical target volume (CTV) motion, acquired from four‐dimensional computed tomography (4D‐CT) information, was used. From the 4D‐CT information, the time‐varying displacement curve of the center of mass of the CTV was obtained for a single breath from maximum inspiration to next maximum inhalation. The phase was divided into 10 regions, from the maximum inspiratory peak to the next peak, which were assigned values of T0 to T90, as shown in Fig. 4 (T0 and T90: maximum inspiration; T50: maximum expiration). The radiation oncologist determined an acceptable phase region based on this curve (eg, T40–T60), with respect to the position to be treated and surrounding critical organs. The treatment planner adjusted the treatment plan to incorporate CTV motion within this phase, then calculated the ratio between the motion within the phase [b (mm) in Fig. 4] and the maximum motion [a (mm) in Fig. 4], in order to derive the threshold level (%) of the respiratory waveform. For example, if the motion within T40–T60 is 1 mm and maximum motion is 6 mm, the threshold level would be 16%. The radiation technologist then input this value as a threshold level on the console of the respiratory gating system. To justify the maximum motion “a(mm)” on the first day of the treatment, the radiation technologist performed X‐ray imaging to determine the maximum inhale and maximum exhale positions. Using anatomical information (preferably a tumor shadow), the maximum motion was measured between two acquired images and the “a(mm)” was verified. If the “a(mm)” exceeded the amplitude obtained from 4D‐CT information, the gate generation level was modified with respect to the difference.

## 3 RESULTS AND DISCUSSION

### 3.A Location and method of sensor installation

**Figure 5:**
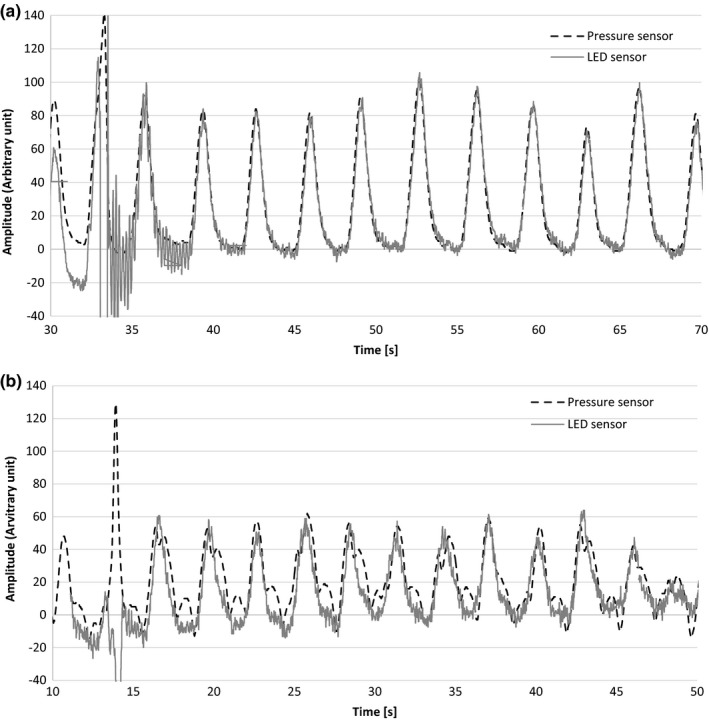
(a**)** Respiratory signals acquired from the pressure sensor and the light‐emitting diode (LED) sensor in the abdominal region in the supine position. (b) Respiratory signals acquired from the pressure sensor and the light‐emitting diode (LED) sensor in the left thorax region in the supine position.

Several locations for installing the sensors were tested for both the supine and the prone positions. The waveforms for both sensors (i.e., pressure sensor and LED sensor), placed in the abdominal region in the supine position, are shown in Fig. 5(a). In most cases, including this one, the signal from the pressure sensor was consistent with the reference signal and LED sensor motion. However, in some locations such as the upper back, cardiac beat was only detected by the pressure sensors. A case in which the sensors were installed on the left thorax in the supine position is shown in Fig. 5(b). This means that it is crucial to select the appropriate pressure sensor installation position in order to acquire an appropriate respiratory signal. In our experience, positions on the thorax near the cardiac region or where body surface motion is very small, such as near the scapular region in the prone position are not appropriate positions for sensor installation. By installing the sensor on the abdominal region where body surface motion is relatively large, it is easy to acquire fine respiratory signals. This information was shared with radiation technologists who normally install the pressure sensors on patients, to avoid mislocation. At the least, the substantially sinusoidal waveform synchronized with the respiratory motion of the patient has to be acquired. In a real clinical situation, it is critical to check this consistency with regard to phase shift. In addition, irregular patient motion and baseline drift of motion should be considered, depending on the patient. Several types of installation instruments, including an in‐house‐manufactured holder, had been tested and validated for stable respiratory signal generation, including belts supplied by the manufacturer.

### 3.B Measurement of system delay time

**Figure 6:**
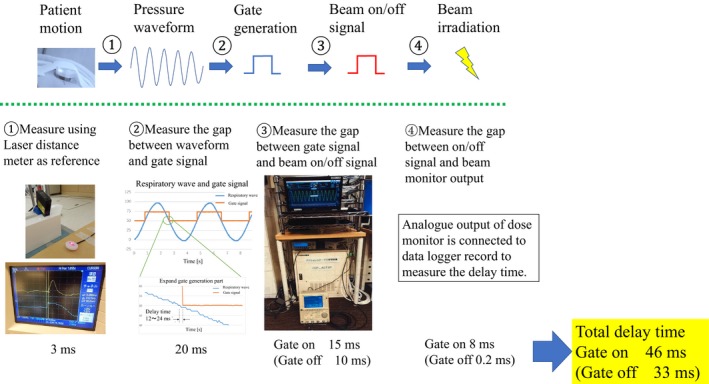
The delay times of each component of the respiratory gating system, and the methods used to measure them.

The measurement results of the delay times of the four components were measured: (a). Actual motion to wave signal generation; (b). Wave signal to gate signal generation; (c). Gate signal to beam on/off signal generation; (d). Beam on/off signal to the beam irradiation. These were 3 ms, 20 ms, 15 ms, and 8 ms (beam on)/0.2 ms (beam off), respectively (Fig. 6). The total delay time of 46 ms (beam on)/33 ms (beam off) was within the tolerance reported in the Physical and Technological Quality Assurance for Particle Beam Therapy guidelines (<100 ms).^16^ It was also within the tolerance value of 100 ms, stated in the TG‐142 report from American Association of Physicists in Medicine.^17^

### 3.C Final flow test

The final flow test was performed with the patient verification system using an actual carbon‐ion beam for the following items: irradiation mode in the R&V system set to “external respiratory gating” in accordance with the planning system, the enabling of a regular patient alignment sequence, successful acquisition of respiratory signals, the enabling of gate generation, the enabling of irradiation with the intended timing, the enabling of rescanning, functioning of interlocks, and the ability to save the respiratory signal/gate/beam on information. The flow test was successful, and the soundness of the system was confirmed. All items listed in the “Respiratory gating system” section were checked and successfully verified.

### 3.D Determination of threshold level to generate gate signal in clinical situations

Herein, we discuss the rationale for threshold level determination for gate signal generation and the limitations of the commissioning process. In this study, we determined whether the system is delivering the correct dose to the target. Specifically, we asked whether setting the gating window at T40–T60 on the system corresponds to the beam‐on when the target is in this phase. Ideally, beam‐on timing should be perfectly matched with T40–T60, however, the respiratory cycle is easily changed in real patients, such that a perfectly reproducible respiratory cycle is hard to acquire in all patients. In addition, our respiratory gating system acts solely through amplitude gating, not on the basis of time information regarding the respiratory cycle. Therefore, we converted the time information to amplitude information, assuming that the movement of the target correlated with body surface movement, in the same manner as that measured by the pressure sensor. In addition to changes in the respiratory cycle, amplitude fluctuates greatly in a real patient. To facilitate safer irradiation, the maximum amplitude (100%) of respiration was determined using normal breathing recorded during the patient alignment time, which was approximately 5 min. This extended observation enabled the radiation technologist to accurately assess the baseline pattern of each patient's respiration. At the stage of treatment beam irradiation, if an extremely small amplitude waveform was observed, manual gate blocking was used to interrupt the beam irradiation. Thus, the irradiation was safely performed within the tolerance amplitude. Certainly, these aspects present limitations in that correlations between target motion and surface motion are not assured. This context must be considered when applying our findings in a clinical setting. A superior method to avoid these limitations is the use of an internal motion gating system. Using fluoroscopic imaging during treatment beam irradiation, the shape of the tumor or surrounding anatomical structure can be used create gate generation of beam‐on/off.

## 4 CONCLUSION

Commissioning of a respiratory gating system involving a pressure sensor for carbon‐ion scanning radiotherapy was successfully performed. The measured delay time of the system was within the tolerance value contained in the system's quality assurance guidelines. All flow tests were executed, and clinical use was initiated.

## CONFLICT OF INTEREST

The authors declare no conflicts of interest.
